# Association between time preference, present-bias and physical activity: implications for designing behavior change interventions

**DOI:** 10.1186/s12889-018-6305-9

**Published:** 2018-12-19

**Authors:** Ruth F. Hunter, Jianjun Tang, George Hutchinson, Susan Chilton, David Holmes, Frank Kee

**Affiliations:** 1 UKCRC Centre of Excellence for Public Health (NI)/Centre for Public Health, Queen’s University Belfast, Institute of Clinical Science B, Royal Victoria Hospital, Grosvenor Road, Belfast, Northern Ireland BT12 6BJ UK; 2 School of Agricultural Economics and Rural Development, Renmin University of China, Beijing, 100872 China; 3 Gibson Institute for Land, Food and Environment, School of Biological Sciences, Queen’s University Belfast, Belfast, Northern Ireland UK; 4 Institute of Global Food Security, School of Biological Sciences, Queen’s University Belfast, Belfast, Northern Ireland UK; 5 Business School—Economics, Newcastle University, Newcastle, England UK

**Keywords:** Physical activity, Behavioral economics, Present-bias, Discount rates, Time preferences, Risk preferences, Public health, UK

## Abstract

**Background:**

The decision to initiate or maintain a healthy habit, such as physical activity involves a trade-off between a short-term cost, such as time and effort, which are commonly identified as barriers to physical activity, and a long-term health benefit. Research suggests that individual time preference may be associated with unhealthy behaviors. However, empirical evidence of this for physical activity is scant. This study investigated the relationship between time preference and physical activity, and how this might influence behavior change.

**Methods:**

Employees (*n* = 176; mean age 42.2 years) who participated in a physical activity intervention were invited to take part in a behavioral economic field experiment. Two economic experiments, using multiple price lists and monetary trade-off tables involving real money choices, were conducted face-to-face with participants to measure the two components of time preference, namely present-bias and discount rate. Together with individual risk preferences, these three variables were jointly estimated by maximum likelihood. These three parameters were expressed as a linear function of the levels of physical activity while controlling for socio-demographic variables within the same maximum likelihood framework.

**Results:**

Those who were present-biased and who had higher discount rates did significantly less physical activity than their patient and non present-biased counterparts. A 3% lower discount rate and 1.14 unit decrement in the present-bias parameter was associated with a 30 min increase of physical activity per week. This negative association was more significant for certain sub-groups, such as younger and married adults and those with higher staff grade and those who have children. Participants who dropped out of the study earlier were more present-biased.

**Conclusions:**

Results demonstrated that discount rate and present-biasedness have a significant impact on physical activity levels. Such concepts have been largely overlooked and underutilized in physical activity interventions. Promising implications include 1) utilizing individuals’ time preferences to better target interventions; 2) taking account of time preferences in the intervention design; 3) interventions attempting to correct for present-biasedness.

**Electronic supplementary material:**

The online version of this article (10.1186/s12889-018-6305-9) contains supplementary material, which is available to authorized users.

## Background

Due to adverse trends in lifestyles factors, such as physical inactivity and sedentary living, healthcare systems globally are facing a budgetary precipice because of an inexorable growth in related conditions such as diabetes. Thus policy makers are calling for a major re-think about how to reverse these trends if we are to have any hope of improving health and sustaining viable healthcare for an aging population [1]. Changing health-related behaviors is not straight forward and there have been a number of high level government reports [2], and influential academic literature that invokes the need to seek innovative and effective interventions by integrating much broader behavioral economics, psychological and socioecological theories [3–5]. If an effective recipe for such interventions can be found, the potential dividend is substantial [6].

However, in practice most of us struggle to lead healthy lives but the question of what role governments should play in encouraging us to do the right thing is contested, and some governments (including the UK, Australia and Norway) have opted for policy “nudges” that change the choice architecture, to make the healthier choices the easier ones [7]. In particular, the UK Government has supported the role of behavioral economics (i.e. the intersection of psychology and economics) [7]. Such approaches have shown potential in public health [8]; however, we know little about how best to utilize and “exploit” such approaches for population level behavior change.

Time preferences, which refers to the extent to which delayed and larger rewards are preferred over immediate and smaller rewards (e.g. Would you choose $100 today or $150 one year from today?), is of particular importance in designing public health interventions [9–16]. In a major contribution to time preference theory, Lowenstein and Prelec (1992) decomposed time preferences into two elements, namely level of discount rates and time-inconsistent discounting [17]. The former refers to the general level of discount rate of a participant (e.g. an individual is said to have a discount rate of 50% if the individual is indifferent between $100 received now and $150 received one year from now) whereas the latter refers to the phenomenon that an individual’s discount rate in the short-term is high and declines as the period of waiting lengthens (e.g. an individual are indifferent between $100 received now and $150 received one year from now but may show strong preference to receive $150 in two years over $100 in a year). This contrasts with time consistent discounting where an individual’s level of impatience is constant over time. Time inconsistency produces what is known as a present-biasedness, an apparent dynamic inconsistency between the preferences of the short-term and long-term self. O’Donoghue and Rabin (1999) recognize that “people grab immediate rewards and avoid immediate costs in a way our long run selves do not appreciate” [18]. Present-biasedness is also referred to as the *self-control* problem [5], the *procrastination* problem [3], or the *short term-self* versus *long term-self* problem [4].

Of significance in this regard is the growing literature on how certain health behaviors are related to an individual’s discount rate and level of present-bias. Research has shown that patient individuals who were more concerned with their future or long-term self’s preferences were more likely to exhibit behaviors associated with positive health consequences—such as physical activity and seeking preventive healthcare—and less likely to procrastinate in adopting healthy behaviors or in exhibiting behaviors associated with negative health consequences from a lack of self-control—such as eating unhealthy foods and smoking [19]. The public health implications of this are very significant as present-biased individuals exhibit problems of self-control and are unlikely to forego present gratification for future health benefit unless present incentives are paid. This may work for one-off or infrequent behaviors, for example vaccination, screening or specialist appointments, but for repetitive daily behavior such as physical activity, engagement of present-biased individuals may wane without a high level of constant incentives. With respect to repetitive behaviors with long term health benefits, present-biased individuals may suffer not only from initiation of the health behavior but also problems of maintenance, both of which are referred to generically as *commitment problems.*

Several recent studies have found that higher discount rate or present-biasedness was negatively associated with participation in physical activity [20–24]. However, parsimonious proxy measures (e.g. accumulation of credit card debt) were used to measure time preference and the two related yet distinct elements of time preference (discount rate and present-biasedness) were not distinguished [20, 23, 24]. Mørkbak et al. (2017) [21] elicited time preference using non-incentive compatible questions. In contrast, Conell-Price and Jamison (2015) [22] reported that more “myopic” individuals (with a bias for the present) exercised more, a conclusion that may be partially explained by exercise providing immediate gratification, in contrast to the view of exercise as a “future-oriented” preventive health behavior, which is standard in the literature. However, their findings may be partially attributable to their measurement of time preference (i.e. a single hypothetical question). Furthermore, none of these studies has utilized objectively measured physical activity. Thus, more in-depth study is necessary to reach a firm conclusion on the association between time preference and physical activity.

Recent reviews have demonstrated evidence of the effectiveness of financial incentives in changing at least some health behaviors [25]. The immediacy of the rewards in such interventions can essentially ‘override’ an individual’s present-bias by incentivizing the participant to undertake the health behavior now, which has health benefits in the future. This immediate reward may be effective in overcoming present-bias for one-off or infrequent compliance behavior but may fail to ensure retention in repetitive behavior unless habit formation quickly takes place or an effective form of compliance management is put in place.

Furthermore, different time discounting behavior (exponential or hyperbolic) may be more or less aligned to different types of preventive health-related behaviors among different types of people, and so in the context of a financial incentive intervention, different incentive structures may be required either to initiate behavior change in the short term or to maintain it in the longer term [26]. For example, physical activity participation may have short term wellbeing gains (e.g. enhanced mood) and losses (e.g. loss of time, fatigue, soreness) and longer-term health benefits whereas smoking cessation has much larger short term wellbeing losses, no short term gains, and coupled with longer term health gains; and it is conceivable to hypothesize that the same discounting function might not align equally well with participants exhibiting these two behaviors.

Therefore, the aim of this study was to investigate the association of time preferences on health behaviors and health behavior change, primarily for physical activity behavior, in the context of a quasi-experimental trial of a workplace financial incentive scheme to promote physical activity [27]. The intervention aimed to promote physical activity, including walking which is one of the most accessible forms of physical activity [28]. We hypothesized that those who maintained regular physical activity in the intervention were less present-biased, more patient individuals (and thus more concerned with long-term health and financial benefits), whereas inactive individuals are more present-biased and impatient and discount both types of future benefits more heavily. To the best of our knowledge, this is the first study to investigate the association by measuring time preference through a series of economic experiments using real money, by jointly estimating discount rates and present-biasedness along with risk preferences [29, 30] and by using an objective measure of physical activity.

## Methods

### The physical activity loyalty (PAL) scheme

The Physical Activity Loyalty (PAL) card scheme (12-week intervention) was a quasi-experimental study where 406 office-based employees from a workplace setting (public sector organization) were each given a loyalty card to monitor their physical activity levels (mainly workplace walking but other forms of physical activity such as running, cycling, use of the gym were included) during office hours, by swiping their card at sensors placed along designated routes within the grounds of their workplace (office buildings based in an extensive natural parkland) [27]. Participants were randomly allocated to either an Incentive or No Incentive group. For the Incentive Group, minutes of physical activity were converted into points and these points could be redeemed for rewards (retail vouchers) sponsored by local businesses. The study collected data on socio-demographic characteristics, objectively measured physical activity (measured as per 100 min of physical activity per week across the 12-week intervention period), retention, self-reported physical activity (Global Physical Activity Questionnaire (GPAQ)) and quality of life (EuroQol 5D). In summary, the study found a trend for ‘modest’ short term increases in physical activity levels and quality of life among those receiving financial incentives [27].

### The field experiments

A random sub-sample of participants from the intervention (Intervention Group) were invited via email (representative sample regarding age, gender and staff grade) to participate in the behavioral economic field experiments after the six month data collection period. A further random sample of participants (in the same workplace) that did not take part in the intervention was invited to participate (Control Group), creating a larger sample size to better identify the discount function which characterizes participants’ time preferences. No further eligibility criteria were applied. A total of 176 participants took part in the field experiments, among which *n* = 95 were from the Intervention Group (PAL-scheme participants) and *n* = 81 from the Control Group (i.e. non PAL-scheme participants). Those expressing an interest in participating were invited to one of the lunchtime sessions. These sessions were facilitated by trained members of the research team, involved 8–16 participants on each occasion and took on average 45 min. Prior to taking part, participants confirmed that they had read and understood the Participant Information Sheet and provided written informed consent. Participants were asked to complete a short questionnaire regarding socio-demographic characteristics. Briefly, 58.5% (*n* = 103) of the sample were female, mean age 42.2 years (*SD* 9.9), 77% (*n* = 135) were single, 67% (*n* = 118) had no children and 16.5% (*n* = 29) were current smokers (see Table 1). The cohort was broadly representative of those employed within the workplace.

**Table 1:** Sample characteristics (*N* = 176)

Characteristics	Grouping criteria	*N* (*%*)	Control Group	Intervention Group	Tests of differences	*p*-value
Gender	Male	73 (41.4*%*)	38	35	*Chi*^*2*^ (1) = 1.83	0.18
	Female	103 (58.5*%*)	73	60		
Age	Mean	42.2	40.5	43.6	*t*(174, 2) = − 2.12	0.04
Staff grade	If staff grade = G5+,G6,G7,DP,SO (higher staff grade)	90 (51.1*%*)	33	57	*Chi*^*2*^ (1) = 6.49	0.01
	If staff grade = EOI,EOII,AO,AA	86 (48.9*%*)	48	38		
Smoker	Current smoker	29 (16.5*%*)	19	10	*Chi*^*2*^ (1) = 5.31	0.02
	Current non-smoker	147 (83.5*%*)	62	85		
Marital Status	Single	135 (76.7*%*)	19	22	*Chi*^*2*^ (1) = 0.00	0.96
	Couple	41 (23.3*%*)	62	73		
Child	Has child/children	58 (33*%*)	30	28	*Chi*^*2*^ (1) = 1.13	0.29
	No child/children	118 (67*%*)	51	67		

Note: Staff-grade was segmented near the mean to have near equal sample sizes

### Elicitation of time preference

To elicit individuals’ time preference, participants were presented with multiple price lists which offered a choice between two monetary amounts (Option A and Option B) (see Additional file 1: Appendix A) [29]. Option A paid a smaller amount whilst Option B offered a larger amount after a longer delay. Each multiple price list consisted of 10 choices between A and B; the sooner amount (Option A) and the delay to receiving Option B remained constant, however progressing down each choice task the interest rate, or reward, for delay increased. According to their time preferences, some participants will accept a smaller reward for a given delay. Six choice tasks corresponding to six different time delays (one month, two months, three months, four months, five months and six months) were used. Each participant was presented with only three time delays randomly chosen from the six to limit the length of sessions since participants were allowed to have up to one hour of lunch time during which experiments were organized. The three chosen time horizons (in a random order) were presented with one-month front end delay (FED, i.e. imposing a delay to both the early and late payment dates) and non-FED, respectively, leading to six choice tasks for each participant. The one month front-end delay treatment was used to avoid the potential problem of extra transaction cost with Option B (which. Includes, credibility of the future money actually being paid etc.). The starting principle for each choice task was £250 (approximately $375) which is comparable to previous similar studies [29].

To ensure that decisions for the tasks were fully incentivized, each participant had a 10% chance of receiving real monetary payments based on their decisions in these tasks. Each participant rolled a 10-sided die and received actual payment if they rolled a 1. A 6-sided die was then rolled to determine which multiple price list was selected to be paid out, followed by a 10-sided die to determine which decision within the list was paid (maximum payment approximately $487). The participant then received the payment that corresponded to their decision in this task. The immediate payments were paid at the end of the sessions by cheques; the delayed payments were paid by posting cheques to the addresses chosen by the participants at the appropriate time delay.

### Elicitation of risk preference

Participants’ risk preferences need to be controlled for to avoid upward-biases to the time preference estimates [29]. To elicit risk preferences, participants were presented with a single multiple price list that consisted of 10 decisions between two lotteries (Option A or Option B) [31]. Each lottery offered a chance to receive a larger or smaller amount of money (See Additional file 1: Appendix B). In the first example, Option A was a lottery consisting of a 10% chance of receiving £140 (~$210) and a 90% chance of receiving £80 (~$120). The difference between Option A and B is that Option B was more risky and had a higher large amount ($300) and a lower small amount ($30). As the participant moved down the 10 decisions in the list the chance of receiving the larger amount in both lotteries increased. For example, a risk neutral participant (choosing solely on expected earnings) would prefer Option A up to choice 5 where they would be indifferent between A and B and from Option 6 onward they would prefer Option B. Furthermore, so-called “risk loving” participants prefer the higher maximum payoff lottery with lower minimum payment (even with smaller expected payoff, i.e. selecting Option B in choices before choice 5) while “risk averse” participants prefer the higher minimum payment lottery with lower maximum payment (even choosing smaller expected payoffs, i.e. selecting Option A in choices after choice 5).

Similar to the time preference tasks, a 10-sided die was rolled to determine if each participant received payment in the risk preference tasks, with participants receiving payment if a 1 was rolled. A second 10-sided die was rolled to determine the pay-out choice and payment was determined by a third throw for the chosen lottery (maximum payment $300). Any payments won in the risk preference tasks were separate to the time preference tasks and were paid by cheques at the end of the field experiment session.

Additional file 1: Appendix F presents a flow diagram to explain the study processes.

### Statistical analyses

The analyses followed a three-step procedure. Firstly, the generalized hyperbolic discount function which nests exponential and hyperbolic discounting and has two components, i.e. present-biasedness and level of discount rate, was jointly estimated with risk preferences by maximum likelihood (ML) (See Additional file 1: Appendix C for a detailed description of the econometric framework). A significantly different from zero present-bias parameter indicates hyperbolic discounting. Data used for the ML estimation included responses on six discounting rate tasks and one risk preference task for each participant from the whole sample (total *n* = 176; *n* = 95 from the Intervention Group and *n* = 81 from the Control Group (i.e. no intervention)). Since each task involved a series of 10 binary choices, this resulted in 12,320 observations. Secondly, the associations between the levels of physical activity and the three parameters (present-biasedness, discount rate and risk preferences) for the Intervention Group were tested by a one-stage approach which estimated the three parameters as a linear function of the levels of physical activity while controlling for socio-demographic variables in the same maximum likelihood framework. Next, it was tested whether the associations between physical activity and time preferences were different across sub-groups of the Intervention Group including age, gender, staff-grade and other household characteristics. Finally, the association between time preferences, risk preferences and trial retention was investigated by constructing an ordinal variable with four categories: 0 = if a participant never used the PAL scheme (0 mins/week throughout the 12-week intervention); 1 = if a participant did some physical activities (> 0 mins/week) during the initial 4 weeks of the intervention but had 0 min for the rest of the intervention; 2 = if a participant did some physical activities in week 1–4 and week 5–8 but had 0 min for the rest of the intervention; 3 = if participants had some physical activities throughout the intervention.

All data were analyzed using Stata version 13. The study was approved by the School of Medicine, Dentistry and Biomedical Sciences Research Ethics Committee, Queen’s University Belfast (Ref: 11/01v1).

## Results

### Hyperbolic versus exponential discount function for the whole sample

Table 2 presents the estimated discount function assuming risk aversion for the whole sample including both Control and Intervention groups. When risk aversion was assumed, discount rate was estimated to be 0.29, substantially smaller than the Figure (0.53) obtained under the risk neutrality condition. Furthermore, present-biasedness was significantly and robustly larger than zero, irrespective of risk aversion ([β=3.23 (SE = 0.57); *p* < 0.01]) or neutrality ([β=3.13 (SE = 0.56); *p* < 0.01]), suggesting that time-inconsistent discounting fitted the data better than exponential discounting.

**Table 2:** Estimations of discount rate and present-biasedness for the whole sample

Parameters	Estimate	Standard error	Lower 95% confidence interval	Upper 95% confidence interval
A. Assuming risk aversion				
Discount rate (*β*)	0.29	0.03	0.22	0.35
Present-biasedness (*α*)	3.23	0.57	2.12	4.35
Risk preference (*γ*)	0.61	0.08	0.45	0.76
*μ*_*RA*_	0.12	0.01	0.09	0.15
*μ*_*DR*_	0.30	0.13	0.04	0.56
Log likelihood	− 6411.17			
Number of observations	12,320			
B. Assuming risk neutrality				
Discount rate (*β*)	0.53	0.04	0.45	0.60
Present-biasedness (*α*)	3.13	0.56	2.03	4.23
*μ*_*DR*_	8.87	0.58	7.73	10.00
Log likelihood	− 6745.38			
Number of observations	12,320			

Note: Standard errors are clustered at the individual level

The estimated discount rates and present-biasedness parameters were used to predict discount rates over time. Additional file 1: Appendix D and E present the summary statistics and fitted discount rates spread over horizons that assume risk aversion and risk neutrality, respectively. It can be seen that time preferences decrease with length of horizons (delays) in both cases. Further, similar to Andersen et al. (2008) [29], time preference estimates decreased dramatically after controlling for risk preference. Therefore, the generalized hyperbolic discount function, controlled for risk preference, was used in subsequent analyses.

### Association between discount rate, present-biasedness, and physical activity behavior of the intervention group

Table 3 shows the association between minutes of objectively measured physical activity and present-biasedness, discount rate, and risk preference for the Intervention Group while controlling for socio-demographic variables. Findings demonstrated that a higher level of physical activity (PAL minutes) was significantly associated with lower discount rates [− 0.091 (0.041); *p* < 0.05], suggesting that a 2.73% point increase in discount rate was associated with 30 min decrease in physical activity per week.

**Table 3:** The association between time preferences and physical activity (100 mins/week) and socio-demographics

Variables	Coefficient	95% CI	Standard error	*t-*value
Dependent variable: Discount rate (*β*)				
*PAL minutes*	− 0.091^b^	[−0.171,-0.012]	0.041	−2.26
*Old*	−0.036	[−0.116,0.044]	0.041	−0.88
*Male*	0.057	[−0.016,0.130]	0.037	1.54
*Household income*	− 0.039	[− 0.091,0.013]	0.026	−1.47
*Single*	−0.138^b^	[−0.263,-0.013]	0.064	−2.17
*Child*	−0.134^c^	[− 0.217,-0.051]	0.042	−3.17
*Own house*	0.097^b^	[0.007,0.186]	0.046	2.12
Constant	0.339^c^	[0.114,0.563]	0.114	2.96
Dependent variable: Present-biasedness (*α*)				
*PAL minutes*	−3.790^c^	[−5.990,-1.591]	1.122	−3.38
*Old*	−2.607^c^	[−3.948,-1.266]	0.684	−3.81
*Male*	1.296	[−0.277,2.869]	0.803	1.61
*Household income*	−0.888^a^	[−1.796,0.020]	0.463	− 1.92
*Single*	−3.619^b^	[−6.697,-0.542]	1.570	−2.31
*Child*	−2.533^c^	[−4.058,-1.008]	0.778	−3.26
*Own house*	5.537^c^	[2.398,8.676]	1.601	3.46
Constant	5.745^b^	[0.959,10.531]	2.442	2.35
Dependent variable: Risk preference (*γ*)				
*PAL minutes*	−0.102^c^	[− 0.177,-0.027]	0.038	−2.68
*Old*	−0.007	[−0.096,0.082]	0.046	−0.16
*Male*	0.003	[−0.069,0.075]	0.037	0.08
*Household income*	0.003	[−0.038,0.044]	0.021	0.16
*Single*	0.065	[−0.029,0.160]	0.048	1.35
*Child*	−0.014	[−0.103,0.076]	0.046	−0.30
*Own house*	0.060	[−0.069,0.189]	0.066	0.91
Constant	0.770^c^	[0.452,1.087]	0.162	4.74
*μ*_*RA*_	0.145^c^	[0.085,0.205]	0.031	4.76
*μ*_*DR*_	0.099	[−0.076,0.274]	0.089	1.11
Log likelihood	− 3371.89			
Number of observations	6650			

Notes: *PAL minutes* is measured as per 100 min of physical activity per week across the 12-week intervention period; *Old* equals 1 if the participants is older than 40, otherwise 0; *Male* takes the value of 1 if the participant is male, 0 otherwise; *Household income* takes five values: 1 (below £15,000), 2 (£15,000 - £29,999), 3 (£30,000 - £ 49,999), 4 (£50,000 - £ 79,999), 5 (£ 80,000 or more); *Single* equals 1 if the participant is single, otherwise 0; *Child* takes the value of 1 if the participant has at least one child, otherwise 0; *Own house* takes the value of 1 if the participant owns a house, otherwise 0; ^a^indicates 10%, ^b^indicates 5% and ^c^indicates 1%. Standard errors are clustered at the individual level

Another important contribution of our analysis is that present-biasedness was also found to be negatively associated with level of physical activity [(− 3.790 (1.122); *p* < 0.01], indicating that a 1.14 unit increase of present-biasedness (self-control problems) was associated with 30 min decrease in physical activity. Finally, we also observed that risk aversion was negatively related to levels of physical activity [− 0.102 (0.038); *p* < 0.01].

Table 3 also reports the association between the socio-demographic variables and discount rates and present-biasedness. It can be seen that those who were single and had child/children had significantly lower discount rates and were less present-biased whereas the individuals who owned houses had significantly higher discount rates and were more present-biased. In addition, those who were older and had higher household income had significantly lower levels of present-bias but did not have significantly different levels of discount rates. Gender was associated with neither present-bias nor the level of discount rate. Finally, none of the socio-demographic variables were significantly associated with risk preferences.

### Association between discount rate, present-biasedness, and physical activity behavior for sub-groups of the intervention group

Analyses showing the association between time preferences and minutes of physical activity for various sub-groups are presented in Table 4. Results showed that lower discount rates were significantly associated with physical activity only among young adults [− 0.141 (0.071); *p* < 0.10], those with a higher staff grade [− 0.143 (0.076); *p* < 0.10], who are married [− 0.185 (0.064); *p* < 0.01] and have child/children [− 0.202 (0.073); *p* < 0.01]. For example, this suggests that an increase of 100 min in a young (age < =40) adult’s physical activity is associated with a 14% decrease in his/her discount rate. It can be inferred that the association of discount rates and physical activity is only significant among some sub-groups but not all. Meanwhile, young adults [− 4.613 (1.826); *p* < 0.05], those with a higher staff grade [− 6.140 (1.610); *p* < 0.01], who are married [− 5.727 (1.373); *p* < 0.01] and have child/children [− 6.773 (3.110); *p* < 0.05] also show a significant negative association between present bias and physical activity.

**Table 4:** The association between time preferences and physical activity (100 mins/week) across sub-groups

	Age		Gender		Staff grade		Marital status		Child	
	Older than 40	Younger than 40	Male	Female	Higher staff grade	Lower staff grade	Single	Married	Has child/children	No child/children
Dependent variable: Discount rate (*β*)										
*Minutes of physical activity*	−0.003 (0.084)	− 0.141^a^ (0.071)	− 0.093 (0.128)	− 0.061 (0.050)	− 0.143^a^ (0.076)	−0.002 (0.090)	0.154 (0.120)	−0.185^c^ (0.064)	− 0.202^c^ (0.073)	0.057 (0.084)
Dependent variable: Present-biasedness (*α*)										
*Minutes of physical activity*	−0.075 (3.071)	− 4.613^b^ (1.826)	−3.529 (2.418)	− 2.255 (8.443)	−6.140^c^ (1.610)	0.147 (2.412)	3.255 (5.430)	−5.727^c^ (1.373)	−6.773^b^ (3.110)	2.021 (3.208)
Dependent variable: Risk preference (*γ*)										
*Minutes of physical activity*	−0.090^b^ (0.040)	−0.033 (0.084)	− 0.061 (0.074)	−0.061 (0.050)	− 0.015 (0.060)	−0.184^c^ (0.043)	− 0.162^b^ (0.064)	−0.039 (0.051)	− 0.029 (0.090)	−0.102^c^ (0.034)
Log likelihood	− 2347.48	− 1172.42	− 1277.73	− 2263.43	− 2127.83	− 1381.80	− 871.20	− 2632.37	−944.04	− 2484.43
Number of observations	4340	2310	2450	4200	3990	2660	1540	5110	1960	4690

Notes: ^a^indicates 10%, ^b^indicates 5% and ^c^indicates 1%. Standard errors are clustered at the individual level

### Association between time preferences and trial retention of the intervention group

Table 5 presents the results of the analyses investigating the influence of time preferences on individuals’ retention throughout the trial. Results demonstrated that those who were more present-biased were less likely to continue in an intervention and dropped out of the study earlier [− 1.819 (0.939); *p* < 0.1]. This is a significant finding as it indicates that present-bias continues to affect retention even after participants have commenced physical activity so its effect is not wiped out by habit formation. Despite the correct sign, no significant relationship was found between trial retention and discount rates [− 0.048 (0.030); *p* > 0.10].

**Table 5:** Association between time preference and trial retention

Variables	Coefficient	95% CI	Standard error	*t-*value
Dependent variable: Discount rate (*β*)				
*Loyalty*	−0.048	[− 0.107,0.012]	0.030	−1.57
*Old*	0.021	[−0.047,0.089]	0.035	0.61
*Male*	−0.026	[−0.096,0.045]	0.036	−0.72
*Single*	−0.167^b^	[−0.306,-0.028]	0.071	−2.36
*Child*	−0.191^c^	[−0.303,-0.078]	0.057	−3.32
*Household income*	−0.030	[−0.074,0.014]	0.023	−1.32
Constant	0.509^c^	[0.187,0.832]	0.164	3.10
Dependent variable: Present-biasedness (*α*)				
*Loyalty*	−1.819^a^	[−3.659,0.022]	0.939	−1.94
*Old*	1.290	[−0.295,2.875]	0.809	1.60
*Male*	−2.471	[−5.483,0.54]	1.537	−1.61
*Single*	−4.089^b^	[−7.405,-0.773]	1.692	−2.42
*Child*	−4.334^c^	[− 7.593,-1.075]	1.663	−2.61
*Household income*	−0.160	[−0.543,0.222]	0.195	−0.82
Constant	11.045^c^	[3.376,18.715]	3.913	2.82
Dependent variable: Risk preference (*γ*)				
*Loyalty*	−0.031^b^	[− 0.061,-0.001]	0.015	−2.05
*Old*	0.032	[−0.036,0.101]	0.035	0.92
*Male*	−0.015	[−0.089,0.058]	0.038	−0.41
*Single*	0.055	[−0.036,0.146]	0.046	1.18
*Child*	−0.014	[−0.098,0.07]	0.043	−0.32
*Household income*	0.002	[−0.038,0.042]	0.020	0.12
Constant	0.789^c^	[0.512,1.067]	0.142	5.57
*μ*_*RA*_	0.142^c^	[0.087,0.197]	0.028	5.07
*μ*_*DR*_	0.129	[−0.075,0.333]	0.104	1.24
Log Likelihood	− 3376.29			
Number of observations	6650			

Note: An ordinal variable *Loyalty* was constructed to indicate retention The four categories included: 0 = if a participant never used the PAL scheme (0 mins/week throughout the 12-week intervention); 1 = if a participant did some physical activities (> 0 mins/week) during the initial 4 weeks but has 0 min for the rest of the intervention; 2 = if a participant did some physical activities in both week 1–4 and week 5–8 but 0 min for the rest of the intervention; 3 = if participants had some physical activities throughout the intervention (week 1–4, week 5–8, week 9–12). Number of participants for each category: Category 0 = 17; Category 1 = 15; Category 2 = 11; Category 3 = 52. ^a^indicates 10%, ^b^indicates 5% and ^c^indicates 1%. Standard errors are clustered at the individual level

## Discussion

### Utilizing time preferences for behavior change

Our results suggest that understanding time preferences has implications for physical activity behavior change and should be utilized to help target interventions more effectively. For example, socio-economic hardships may shorten people’s time horizons and it has been suggested that low socio-economic position (SEP) might also relate to present time orientation and impulsivity [32]. These groups may benefit from interventions such as financial incentive-based interventions that help those with high time discount rates to initiate healthy behaviors with long-term benefits [33] or interventions that explicitly account for differing time preferences. For example, time preference rates have been used to set realistic weight-loss goals in overweight and obese populations [34], and for tailoring communication for promoting healthy eating [35]. Such interventions can frame messages to heighten the focus on short term benefits, for example, improved mood and increased energy.

### Changing and modifying the effects of time preferences

Changing temporal perspectives that lead to sub-optimal health choices (i.e. time-inconsistent or present-bias preferences (a behavioral economics concept, compared to the neo-classical concept of time-consistent preferences)) is an emerging topic in public health research and may have important implications for public health. Time consistent discounting implies that, in theory, agents have perfect foresight into the future evolution of their discount rates and would therefore employ pre-commitment strategies to enable themselves to commit to an optimal plan. We found that this was not supported by our study because participants manifested time-inconsistent discounting. Laibson (1997) developed the idea of “commitment contracts” to enable individuals with time inconsistent preferences to make alternative, optimal choices about current behavior [36]. Recent studies suggest that time preferences can be changed or modulated by therapeutic cognitive, behavioral, or structural environmental manipulation [37]. It is shown that many people joining gyms choose fixed term membership which can work out to be much more costly than paying per visit [38]. They also failed to cancel monthly membership long after use of the gym had ceased. This appears to provide evidence that new participants were using monthly membership as a means of commitment management in an attempt to increase their physical activity participation because they know they are overconfident in their future self control abilities.

Shifting preferences to favor long-term outcomes requires either suppressing or ignoring participant’s desire for the immediate reward or down-regulating its value (i.e. shifting attentional resources from “now” to later). In particular, training cognitive skills such as attention, working memory, and executive functioning is believed to be effective in changing time preference biases [39]. Through the simple reframing of a message of a classical discounting choice as “something now but nothing later” versus “nothing now but more later” has been shown to decrease apparent time preferences [39]. It has been argued that knowledge of self-control problems can modify effects of present bias [18]. The Temporal Self-regulation Theory, which suggests that those individuals with stronger executive control are more able to do physical activity consistently, is a useful model to underpin such interventions [18]. Furthermore, contingency management interventions [40] provide incremental reinforcement (in the form of vouchers or other tangible rewards) that is contingent upon repetition of the desired behavior. It forces participants to make a choice between “using now” and “earning no reward later”.

### Implications for public health

Our findings, and those of others, suggest that time preferences have significant implications for developing interventions to increase physical activity and improve public health. Three promising implications include 1) utilizing individuals’ time preferences to better target behavior change interventions for hard to reach sub-groups with present-biasedness/or high discounting rates; 2) taking account of time preferences in the design of the intervention, for example, in setting short-term goals tailored by individual’s time preferences; 3) devising interventions that attempt to correct for present-biasedness. Such interventions could help bridge the gap between previous interventions which tended to put too much weight on costs and benefits that are immediate and too little on those that are delayed [8]. Interventions of this kind could potentially improve multiple health outcomes as time preferences are also associated with health-related behaviors other than physical activity, such as eating habits and smoking [32]. Indeed, these approaches should be more suitable for changing physical activity behavior given that the behavior already provides short-term positive benefits such as improved mood and wellbeing.

### Implications for future research

Despite a growing number of trials investigating the association between time preferences for physical activity behavior change and an emerging literature on time preference interventions [39, 41], few studies have, by design, harnessed the power of time preferences within bespoke public health interventions. Theory [41] and a small number of simulated “experiments” [42, 43] suggest that time preference parameters can mediate the transmission or adoption of health behaviors. In addition, O’Donoghue and Rabin (1999) [18] asserted that where some present-biased individuals foresee their self-control problems (referred to *sophistication*) while others fail to foresee the problems (referred to *naïve*), the effects of this bias can be lessened or increased. Individuals characterized by a certain phenotype may respond differently to commitment contracts, thus future studies should take this into account.

### Strengths and limitations

Key strengths include the use of an objective measure of physical activity and economic experiments with real monetary payments which have stronger associations with health behaviors than hypothetical methods and self-reported measures [11]. Although, this objective measure has not been validated to date, it was supplemented with the GPAQ which is a well-validated self-report measure. Further, the estimation of discount rates and present-biasedness were controlled for risk preferences by joint estimations to eliminate upward biases caused by separate estimations of these variables [29]. However, as this was cross-sectional data we were unable to establish the stability of these measures. Substantial incentives in the form of cash presented in these elicitation processes ensured that participants responded carefully and truthfully in comparison to hypothetical elicitations where the considerable effort required in the process is not related to real rewards. However, results from the sub-group analyses should be interpreted with caution due to the smaller sample size, and may not be generalizable to physical activity outside of the workplace setting and in the wider population. Our study utilizes cross-sectional data; further research analyzing longitudinal data will be required in order to infer causality. Further, although the intervention was designed to promote physical activity, the majority of participants undertook walking and so the results may not be generalizable to other forms of physical activity.

## Conclusion

The decision to initiate or maintain a healthy habit, such as physical activity (in this case workplace physical activity) involves a trade-off between a short-term cost, such as time and effort, which are commonly identified as barriers to physical activity, and a long-term health benefit. Understanding individuals’ time preferences can therefore inform appropriate interventions to address health behaviors. Results demonstrated that discount rate and present-biasedness were negatively associated with physical activity levels and the impact is more significant for specific sub-groups, for example, younger adults. Present-biasedness was shown to be related to trial retention which is at the core of time inconsistent preferences and resolving the different preferences of the short term and long term self. Our research suggests that future physical activity interventions should take account of individuals’ time preferences including present-biasedness and incorporate these into intervention design, such as goal setting, incentive levels and framing of messages. Failure to identify present-biased, impatient individuals, who are the most likely to form sub-optimal (self) commitment plans due to overconfidence, procrastination and self-control problems, will lead to interventions that continue to be (largely) unsuccessful in the long term. We argue that behavioral economic concepts such as discount rate and present-bias have been largely overlooked and underutilized to date in designing public health interventions and could shed light on important factors contributing to uptake and maintenance of healthy behaviors.

## Additional file

Additional file 1: Appendix A-F. (DOCX 67 kb)

## Abbreviations

FED: Front end delay
GPAQ: Global physical activity questionnaire
ML: Maximum likelihood
PAL: Physical activity loyalty
SD: Standard deviation
SEP: Socio-economic position
UK: United Kingdom
