# Supplementary material for: Association between time preference, present-bias and physical activity: implications for designing behavior change interventions
Source: BMC Public Health. 2018 Dec 19;18:1388. doi: 10.1186/s12889-018-6305-9 (PMC6300013; doi:10.1186/s12889-018-6305-9)
Supplement: Supplementary file 1 — Appendix A-F. (DOCX 67 kb) [file 12889_2018_6305_MOESM1_ESM.docx]

| **Appendix A: Example of time preference choice tasks**  Without a one-month front-end delay (time delay = six months)   \| Choice \| Option A: Amount received today \| Option B: Amount received in 6 months \| Annual Interest Rate (%) \| Circle Your Choice: \| \| --- \| --- \| --- \| --- \| --- \| \| 1 \| £250.00 \| £256.37 \| 5 \| A or B \| \| 2 \| £250.00 \| £262.98 \| 10 \| A or B \| \| 3 \| £250.00 \| £269.83 \| 15 \| A or B \| \| 4 \| £250.00 \| £276.94 \| 20 \| A or B \| \| 5 \| £250.00 \| £284.30 \| 25 \| A or B \| \| 6 \| £250.00 \| £291.93 \| 30 \| A or B \| \| 7 \| £250.00 \| £299.83 \| 35 \| A or B \| \| 8 \| £250.00 \| £308.01 \| 40 \| A or B \| \| 9 \| £250.00 \| £316.47 \| 45 \| A or B \| \| 10 \| £250.00 \| £325.23 \| 50 \| A or B \|   With a one-month front-end delay (time delay = six months) | | | | |
| --- | --- | --- | --- | --- | --- | --- | --- | --- | --- | --- | --- | --- | --- | --- | --- | --- | --- | --- | --- | --- | --- | --- | --- | --- | --- | --- | --- | --- | --- | --- | --- | --- | --- | --- | --- | --- | --- | --- | --- | --- | --- | --- | --- | --- | --- | --- | --- | --- | --- | --- | --- | --- | --- | --- | --- | --- | --- | --- | --- |
| Choice | Option A: Amount received in 1 month | Option B: Amount received in 7 months | Annual Interest Rate (%) | Circle Your Choice: |
| 1 | £250.00 | £256.37 | 5 | A or B |
| 2 | £250.00 | £262.98 | 10 | A or B |
| 3 | £250.00 | £269.83 | 15 | A or B |
| 4 | £250.00 | £276.94 | 20 | A or B |
| 5 | £250.00 | £284.30 | 25 | A or B |
| 6 | £250.00 | £291.93 | 30 | A or B |
| 7 | £250.00 | £299.83 | 35 | A or B |
| 8 | £250.00 | £308.01 | 40 | A or B |
| 9 | £250.00 | £316.47 | 45 | A or B |
| 10 | £250.00 | £325.23 | 50 | A or B |

| **Appendix B: Example of risk preference choice tasks** | | | |
| --- | --- | --- | --- |
| Choice | Option A | Option B | Circle you choice: |
| 1 | £140 if die is 1  £80 if die is 2-10 | £200 if die is 1  £20 if die is 2-10 | A or B |
| 2 | £140 if die is 1-2  £80 if die is 3-10 | £200 if die is 1-2  £20 if die is 3-10 | A or B |
| 3 | £140 if die is 1-3  £80 if die is 4-10 | £200 if die is 1-3  £20 if die is 4-10 | A or B |
| 4 | £140 if die is 1-4  £80 if die is 5-10 | £200 if die is 1-4  £20 if die is 5-10 | A or B |
| 5 | £140 if die is 1-5  £80 if die is 6-10 | £200 if die is 1-5  £20 if die is 6-10 | A or B |
| 6 | £140 if die is 1-6  £80 if die is 7-10 | £200 if die is 1-6  £20 if die is 7-10 | A or B |
| 7 | £140 if die is 1-7  £80 if die is 8-10 | £200 if die is 1-7  £20 if die is 8-10 | A or B |
| 8 | £140 if die is 1-8  £80 if die is 9-10 | £200 if die is 1-8  £20 if die is 9-10 | A or B |
| 9 | £140 if die is 1-9  £80 if die is 10 | £200 if die is 1-9  £20 if die is 10 | A or B |
| 10 | £140 if die is 1-10 | £200 if die is 1-10 | A or B |

**Appendix C: Econometric Framework**

Following Holt and Laury (2002), we assume the utility function to show constant relative risk aversion (CRRA) as described in (1):

$U\left( M \right)=\frac{{(\omega+M)}^{1-r}}{1-r}$ (1)

where $r$ is the CRRA coefficient and *M* the payoff. $\omega$ represents the daily background consumption. Following Harrison et al. (2010), $\omega$ is taken as the population-level daily consumption of private non-durable goods (£15).

First, we constructed a likelihood function for risk preference choice tasks. The expected utilities of each of the 10 lotteries in the Risk Preference Choice Task (Appendix A.2) is expressed as follows:

${EU}_{A}=p\left( M_{1} \right)\times\frac{{(\omega+M_{A1})}^{1-r}}{1-r}+p\left( M_{2} \right)\times\frac{{(\omega+M_{A2})}^{1-r}}{1-r}$ (2)

${EU}_{B}=p\left( M_{1} \right)\times\frac{{(\omega+M_{B1})}^{1-r}}{1-r}+p\left( M_{2} \right)\times\frac{{(\omega+M_{B2})}^{1-r}}{1-r}$ (3)

where $p\left( M_{1} \right)$ and $p\left( M_{2} \right)$ are the probabilities for the payoffs, $M_{A1}$ ($M_{B1}$) and $M_{A2} (M_{B2})$are the high and low payoffs in Option A(B), respectively. ${EU}_{A}$ and ${EU}_{B}$ are expected utilities for lottery *A* and *B*, respectively.

The index $\nabla EU$ measures the differences of the expected utility between Option A and B. Thus, the probability of an individual choosing Option A, ${Pr}_{i}^{R}\left( A \right)$, is represented as:

${Pr}_{i}^{R}\left( A \right) =\Phi\left( \nabla EU \right)=\Phi({EU}_{A}-{EU}_{B})/\mu_{RA}$ (4)

where ${Pr}_{i}^{R}\left( A \right)$ refers to the probability of choosing Option A in Appendix A.2, $\Phi$ represents a cumulative probability distribution function over $\nabla EU$, and $\mu_{RA}$ is a structural noise parameter (Wilcox, 2008). The likelihood function for the risk preference choice tasks is constructed as in (5).

$lnL^{R}(\gamma,\mu;y,\omega,X)=\sum_{i=1}^{10} ((ln{(Pr}_{i}^{R}\left( A \right)|y_{i}=A)+ln(1-{Pr}_{i}^{R}\left( A \right)|y_{i}=B))$ (5)

where $y_{i}=A(B)$ indicates choice of Option A(B) in the risk preference task *i*. The risk preference $r$ is allowed to be a linear function of the characteristics in vector *X* which is constant by default and can be defined as a set of socio-demographic variables including age, gender, income, household characteristics, level of physical activity etc.

Next, we constructed the likelihood function for the time discounting tasks (for an example see Appendix A). Participants were asked to make a choice between a recent reward $M_{t}$paid at time $t$ and a larger amount $M_{t+\tau}$ paid at a future time $t+\tau$, where $\tau$ is the horizon for delivery of the later reward. The discounting function ${1/(1+\alpha t)}^{\frac{\beta}{\alpha}}$equates the future reward $M_{t+\tau}$, to the recent reward $M_{t}$.

$M_{t}={1/(1+\alpha t)}^{\frac{\beta}{\alpha}}\times M_{t+\tau}$, (6)

where ${1/(1+\alpha t)}^{\frac{\beta}{\alpha}}$ is a generalized hyperbolic discount function proposed by Loewenstein and Prelec (1992) and has two components: discount rate ($\beta$) and present-biasedness ($\alpha$). This discount function is employed because it was identified to have the best-fit among a series of hyperbolic discount functions (i.e. Mazur-hyperbolic, Quasi-hyperbolic, Weibull-hyperbolic, and generalized-hyperbolic) analysed using the same dataset (details see Tang et al., 2016). The parameter $\alpha$ is an indicator of hyperbolic against exponential discount functions. If $\alpha$ is close to 0, the discount function collapses to the exponential discounting function $exp(-\delta t)$. If $\alpha$>0, the discount rates decrease over time, indicating the presence of present-bias. The level of $\alpha$ determines the steepness of the decline. Assuming ${PV}_{A}$ and ${PV}_{B}$ are discounted utilities of Option A and B, respectively, we have:

${PV}_{A}=\frac{{(\omega+M_{t})}^{1-r}}{1-r}+ {1/(1+\alpha t)}^{\frac{\beta}{\alpha}}\times\frac{\omega^{1-r}}{1-r}$ (7)

${PV}_{B}=\frac{\omega^{1-r}}{1-r}+{1/(1+\alpha t)}^{\frac{\beta}{\alpha}}\times\frac{{(\omega+M_{t+\tau})}^{1-r}}{1-r}$ (8)

${PV}_{A}$ and ${PV}_{B}$ are the payments for Option A and B, respectively.We follow Laury et al. (2012) and define ${Pr}_{i}^{D}\left( A \right)$ as the probability of choosing Option A in the time preference tasks.

${Pr}_{i}^{D}\left( A \right) =\Phi\left( \nabla PV \right)={\Phi(PV}_{A}-{PV}_{B})/\mu_{DR}$ (9)

where $\nabla PV$ refers to the differences of utilities between Option A and B. $\mu_{DR}$ is similar to the $\mu_{RA}$ in (5), and $\Phi$ represents standard cumulative normal distribution function.

Then the conditional log-likelihood function can be written as:

$lnL^{D}(\alpha, \beta,r,\mu_{RA},\mu_{RA};y,\omega,X)=\sum_{i=1}^{60} ((ln{(Pr}_{i}^{D}\left( A \right)|y_{i}=A)+ln(1-{Pr}_{i}^{D}\left( A \right)|y_{i}=B))$ (10)

where $y_{i}=A(B)$ refers to the choice of A(B) in the time preferences choice task *i*. The vector *X* is the same as in (5).

Following Andersen et al. (2008) and Harrison et al. (2014), the joint likelihood of the time preference and risk preference is as follows:

$lnL\left( \delta,r,\mu;y,\omega,X \right)= ln\left( L^{R} \right)+ln\left( L^{D} \right)$ (11)

This equation (11) can be maximized using standard numerical methods.

**References**

Andersen, S., Harrison, G.W, Lau, M.I., Rustrom, E.E., 2008. Eliciting time and risk preferences. Econometrica 76, 583–618.

Andersen, S., Harrison, G.W., Lau, M.I., Rutström, E.E., 2014. Discounting behavior: A reconsideration. European Economic Review 71, 15–33.

Harrison, G.W., Lau, M.I., Rustrom, E.E., 2010. Individual discount rates and smoking: Evidence from a field experiment in Denmark. Journal of Health Economics 29, 708–719.

Holt, C.A., Laury, S.K., 2002. Risk aversion and incentive effects. American Economic Review 92, 1644–1655.

Laury, S.K., McInnes, M.M., Swarthout, J.T., 2012. Avoiding the curves: Direct elicitation of time preferences. Journal of Risk and Uncertainty 44, 181–217.

Loewenstein, G., Prelec, D., 1992. Anomalies in intertemporal choice: Evidence and interpretation. Quarterly Journal of Economics 107, 573–597.

Tang, J., Hutchinson, G., Chilton, S.M., Hunter, R.F., Lau, M., Kee, F., 2017. Exponential or hyperbolic? Identifying and testing the predictive power of time preference over unhealthy behaviours. *Submitted*.

Wilcox, N.T., 2008. Stochastic models for binary discrete choice under risk: A critical primer and econometric comparison. In: Cox, J., Harrison, G.W., (Ed). Risk aversion in experiments. Volume 12. Bingley, UK: Emerald, Research in Experimental Economics.

| **Appendix D: Summary statistics of estimated discount rates across time (whole sample)** | | | | |
| --- | --- | --- | --- | --- |
| Discount rates | Estimate | S.E. | Lower 95% confidence interval | Higher 95% confidence interval |
|  |  |  |  |  |
| Assuming risk neutrality | | | | |
| Horizons |  |  |  |  |
| 1 day | 68.8% | 6.5% | 56.1% | 81.4% |
| 3 days | 68.0% | 6.3% | 55.8% | 80.3% |
| 7 days | 66.6% | 5.9% | 55.0% | 78.2% |
| 2 weeks | 64.3% | 5.4% | 53.8% | 74.8% |
| 1 month | 59.5% | 4.4% | 51.0% | 68.1% |
| 2 months | 52.7% | 3.2% | 46.4% | 58.9% |
| 3 months | 47.5% | 2.5% | 42.5% | 52.4% |
| 6 months | 37.2% | 1.7% | 33.8% | 40.6% |
| 9 months | 31.1% | 1.5% | 28.1% | 34.0% |
| 12 months | 26.9% | 1.4% | 24.2% | 29.6% |
|  | | | | |
| Assuming risk averse | | | | |
| Horizons |  |  |  |  |
| 1 day | 33.2% | 4.3% | 24.8% | 41.6% |
| 3 days | 32.9% | 4.2% | 24.6% | 41.1% |
| 7 days | 32.2% | 4.1% | 24.2% | 40.2% |
| 2 weeks | 31.2% | 3.9% | 23.6% | 38.7% |
| 1 month | 29.0% | 3.4% | 22.3% | 35.8% |
| 2 months | 25.9% | 2.9% | 20.2% | 31.6% |
| 3 months | 23.5% | 2.6% | 18.4% | 28.5% |
| 6 months | 18.7% | 2.0% | 14.8% | 22.6% |
| 9 months | 15.7% | 1.7% | 12.4% | 19.0% |
| 12 months | 13.7% | 1.5% | 10.8% | 16.6% |

**Appendix E: Estimated discount rates over time**

**Appendix F. CONSORT Flow diagram**

Assessed for eligibility (n=644)

Randomised (n=406)

**Allocation**

Allocated to No Incentive Group (n=207)

Allocated to Incentive Group (n=199)

**12-week intervention period**

Objective PA measure (n=207)

Self-report completion (n=175)

Objective PA measure (n=199)

Self-report completion (n=168)

**Follow-up**

**12 weeks**

**Follow-up**

**six months**

Objective PA measure (n=207)

Self-report completion (n=175)

Objective PA measure (n=199)

Self-report completion (n=168)

**Behavioural economic field experiments**

**(elicitation of time preference task and elicitation of risk preference task**

**six months**

Invited to participate in field experiments (n=199)

Completed field experiments (n=95)

Employees who did not participant in the study to date were invited to participate in field experiments; n=81completed the field experiments
